# Tumor Immune Microenvironment and Checkpoint Inhibition in Clear Cell Ovarian Carcinoma: Bridging Tumor Biology and Clinical Application in Immunotherapy

**DOI:** 10.3390/cimb47090726

**Published:** 2025-09-05

**Authors:** Fulvio Borella, Giulia Capella, Stefano Cosma, Niccolò Gallio, Federica Gavello, Alberto Revelli, Domenico Ferraioli, Jessica Cusato, Isabella Castellano, Paola Cassoni, Luca Bertero

**Affiliations:** 1 Gynecology and Obstetrics 1U, Department of Surgical Sciences, University of Turin, 10126 Turin, Italy; stefano.cosma@unito.it; 2 Pathology Unit, Department of Medical Sciences, University of Turin, 10126 Turin, Italy; giulia.capella@unito.it (G.C.); isabella.castellano@unito.it (I.C.); paola.cassoni@unito.it (P.C.); luca.bertero@unito.it (L.B.); 3 Gynecology and Obstetrics 2U, Departments of Surgical Sciences, University of Turin, 10126 Turin, Italy; niccolo.gallio@edu.unito.it (N.G.); federica.gavello@unito.it (F.G.); alberto.revelli@unito.it (A.R.); 4 Department of Gynecology, Léon Bérard, Comprehensive Cancer Centre, 69008 Lyon, France; domenico.ferraioli@lyon.unicancer.fr; 5 Laboratory of Clinical Pharmacology and Pharmacogenetics, Department of Medical Sciences, University of Turin, 10149 Turin, Italy; jessica.cusato@unito.it

**Keywords:** ovarian clear cell cancer, ovarian cancer, immunotherapy, clear cell

## Abstract

Clear cell ovarian carcinoma is a rare and aggressive histologic subtype of epithelial ovarian cancer, characterized by a chemoresistant phenotype and distinct immunogenomic features. Despite early-phase trials showing a limited response to immune checkpoint inhibitors (ICIs), emerging evidence reveals a biologically diverse tumor immune microenvironment, with implications for the efficacy of immunotherapies. Preclinical studies highlight paradoxical associations between immune infiltration and prognosis, as well as genomic drivers—including KRAS, MYC, PI3KCA, TP53, PTEN, and ARID1A—that shape immune evasion and checkpoint ligand expression. Clinically, ICI monotherapy yields modest benefit, while combination regimens—particularly dual checkpoint blockade and targeted co-inhibition—offer improved outcomes. Biomarkers such as PD-L1 CPS ≥ 1%, ARID1A mutations, elevated tumor mutational burden, and PIK3CA alterations emerge as promising predictors of therapeutic response. This review integrates current preclinical and clinical data to propose a precision immunotherapy framework tailored to the immunogenomic landscape of clear cell ovarian carcinoma.

## 1. Introduction

Ovarian cancer (OC) ranks as the eighth-most-common malignancy among women worldwide, representing a major global health concern. In 2020, there were an estimated 313,959 newly diagnosed cases, accounting for 1.6% of all cancers, and 207,252 deaths, making up 2.1% of cancer-related mortality among women [1]. Despite ongoing advances in diagnostic and therapeutic approaches, the prognosis for OC remains poor.

Within this spectrum, ovarian clear cell carcinoma (OCCC) is recognized as a rare and distinct histological subtype of epithelial ovarian cancer (EOC), comprising approximately 10% of all OC cases. Characterized by unique morphological and molecular traits, OCCC exhibits a distinct clinical course compared to other EOC subtypes. A notable feature of OCCC is its geographic and ethnic variation in incidence, with significantly higher prevalence reported in East and Southeast Asian populations, ranging from 10% to 30%, compared to 5% to 12% in Western countries [2,3]. These epidemiological discrepancies have prompted extensive investigation into potential contributing factors. Hypotheses include environmental influences (e.g., dietary patterns, exposure to toxins), genetic susceptibility (e.g., germline mutations or polymorphisms), and distinct oncogenic pathways driven by inflammation- or endometriosis-related processes [4,5]. The exact etiopathogenetic mechanisms behind the ethnic disparity remain to be fully elucidated.

Compared with high-grade serous ovarian carcinoma (HGSOC)—the most common and aggressive ovarian cancer subtype—OCCC exhibits distinctive clinical characteristics. These include a younger median age at diagnosis, frequent association with endometriosis, lower response rates to chemotherapy, and specific molecular alterations [6,7].

Most cases of OCCC are diagnosed at an early stage and are associated with relatively favorable survival outcomes. Conversely, advanced-stage OCCC presents a significant therapeutic challenge. These tumors frequently demonstrate intrinsic resistance to platinum-based chemotherapy, a cornerstone of EOC treatment, which contributes to poor prognosis and limited treatment options in this setting [8].

In light of OCCC’s chemoresistant profile and the limited efficacy of standard therapeutic regimens—particularly in advanced-stage disease—novel strategies are urgently needed. Growing evidence underscores the role of the tumor immune microenvironment (TIME) in shaping OCCC’s behavior and therapeutic response. Recent studies have revealed immunogenic features, including AT-rich interaction domain-containing protein 1A (ARID1A) mutations, programmed death-ligand (PD-L1) expression, and CD8^+^ T cell infiltration, suggesting that a subset of OCCC may benefit from immunotherapeutic interventions [9,10]. Immune checkpoint blockade, particularly targeting the PD-1/PD-L1 axis, has emerged as a promising approach, with early clinical data indicating potential benefit in select patient cohorts. Understanding the complex interplay between TIME components and molecular alterations is therefore critical to refining patient selection and improving immunotherapy outcomes in OCCC [9,10]. This review aims to summarize the latest developments in the field of OCCC immunoncology and to discuss immune checkpoint inhibitors (ICIs) potential for this subset of OC.

## 2. PD-L1 Expression in Clear Cell Ovarian Carcinoma

PD-L1 is a transmembrane protein that plays a pivotal role in tumor immune evasion. By binding to its receptor PD-1 on activated T cells, PD-L1 transmits inhibitory signals that suppress T cell proliferation, cytokine production, and cytotoxic activity. This interaction contributes to the establishment of an immunosuppressive TIME, allowing malignant cells to escape immune surveillance. Elevated PD-L1 expression has been observed across various cancer types, including non-small cell lung carcinoma, cervical cancer, melanoma, as well as OCCC. It is associated with poor prognosis and resistance to conventional therapies [11,12,13,14,15]. Therapeutic blockade of the PD-1/PD-L1 axis using ICIs has demonstrated significant clinical benefit, restoring antitumor immunity and improving survival outcomes in select patient populations.

To gain a deeper understanding of immune cell infiltration and checkpoint dynamics in OCCC, a multiplex immunohistochemistry analysis was conducted in Canada on 162 tumor specimens. Antibodies were sourced from Biocare Medical (Pacheco, CA, USA) and distributed by Inter Medico (Markham, ON, Canada). This comprehensive profiling quantified various immune populations—including pan-T (CD3^+^), cytotoxic T (CD8^+^), T helper (Th) (CD3^+^CD8^−^), regulatory T (FoxP3^+^), B cells (CD20^+^), and macrophages (CD68^+^)—alongside key immune checkpoints such as PD-1, PD-L1, and indoleamine 2,3-dioxygenase 1 (IDO1). Tissue microarray sections were scanned with the Vectra multispectral imaging system and analyzed using PerkinElmer’s InForm software for tissue segmentation and immune marker quantification. Three independently trained algorithms identified chromogen-positive pixels, and their average counts were used for analysis. Immune cell abundance (CD8^+^, FoxP3^+^, CD3^+^, CD68^+^, CD20^+^) and immune checkpoint protein expression (PD-L1, PD-1, IDO-1) were quantified by summing positive signals in both epithelial and stromal compartments. Unexpectedly, elevated infiltration of cytotoxic and Th cells, along with macrophages, was associated with worse disease-free, recurrence-free, and overall survival (OS). In contrast, higher expression levels of PD-L1 and IDO1 were correlated with more favorable clinical outcomes, suggesting a potentially protective prognostic role [16].

Further evidence from a retrospective analysis of 125 cases in Japan reinforced the prognostic relevance of PD-L1. Immunohistochemical analysis revealed PD-L1 positivity in 86.4% of patients’ evaluation (antibody clone E1L3N, cut-off 5%), with expression significantly associated with diminished chemotherapy response and reduced progression-free (PFS) and OS. Notably, PD-L1 retained independent prognostic value after adjusting for clinical covariates and showed no association with TIL density or mismatch repair MMR status, supporting its role as a standalone biomarker [17].

Additional insights emerged from a Chinese cohort of 152 OCCC patients, where paired analyses across primary, recurrent, and metastatic lesions illuminated the dynamic nature of PD-L1 expression using the 22C3 pharmDx assay. Positivity rates were varied by scoring system—22.4% with tumor proportion score (TPS, cut-off 1%) and 38.8% with combined positive score (CPS), with 21.7% surpassing a high-expression threshold (CPS ≥ 10). Crucially, PD-L1 expression was sustained or elevated in recurrent lesions and associated with platinum resistance and aggressive clinicopathological features, including advanced FIGO stage and distant metastasis. These findings support PD-L1 as both a prognostic and predictive biomarker, especially with CPS scoring. Recurrent and metastatic lesions proved suitable for PD-L1 assessment, while lymphatic metastases were deemed unreliable [18].

Exploring spatial immune architecture, comparative evaluation of tumor and adjacent nonneoplastic tissues in 18 OCCC Chinese patients revealed divergent immune marker distribution. For the determination of PD-L1, (1:500, Servicebio, GB11339) was used; positivity was considered with CPS ≥ 1. While PD-1 and PD-L1 lacked prognostic relevance in either compartment, CD8^+^ cytotoxic T cells were markedly enriched in nontumor stroma. A differential of >70 cells/mm^2^ between nontumor-infiltrating lymphocytes and intratumoral TILs predicted poorer PFS. Elevated CD8^+^ density in peritumoral stroma further correlated with unfavorable outcomes, suggesting that immune localization, rather than abundance alone, may be critical to prognosis [19].

Finally, an integrated clinicopathologic analysis of 76 OCCC cases in Taiwan examined interactions among PD-L1 expression, CD8^+^ TILs, and major histocompatibility complex class I (MHC-I) status. Immunohistochemistry for PD-L1 was performed using an SP263 antibody (prediluted; Roche Ventana, Tucson, AZ, USA); TPS ≥ 1% and CPS ≥ 1 were defined as positive. Nearly half of the tumors showed MHC-1 class I loss, inversely associated with CD8^+^ infiltration—especially in PD-L1^+^ cases. Although PD-L1 positivity often aligned with higher TIL density, elevated CD8^+^ levels paradoxically predicted poorer outcomes. These patterns suggest that PD-L1^+^ immune-active profiles may reflect immune evasion, such as MHC-1 downregulation, rather than effective antitumor immunity [20].

Complementing these findings, a separate retrospective investigation spanning 46 paraffin-embedded specimens of pure ovarian and uterine CCCs assessed PD-1-associated TILs, PD-L1 positivity, and CPS alongside clinical staging, endometriosis history, and survival data. For PD-L1 staining, Ventana SP263 rabbit monoclonal antibody was used; positivity of PD-L1 was considered for CPS ≥ 1. Most cases presented at FIGO stage I/II (63.0%) [21]. PD-L1 CPS ≥ 1 was observed in 34.3% of ovarian and 60% of uterine tumors, with PD-1^+^ lymphocyte infiltration in 39.4% and 80%, respectively. Among ovarian cancers, PD-L1 CPS ≥ 1 was significantly associated with higher disease stage (*p* = 0.03), although PD-1 expression showed no stage-specific variation (*p* = 0.227). No such stage-dependent associations were observed within uterine tumors. Multivariate analyses did not reveal additional significant correlations, and PD-L1 CPS status was not prognostic of survival outcomes in the ovarian CCC cohort (*p* = 0.79) [21].

Recent studies have begun to shed light on the immunogenomic interplay between MMR deficiency and PD-L1 expression in OCCC. In one immunohistochemical analysis involving 28 cases in Iran, researchers examined PD-L1 expression both within tumor cells and the surrounding peritumoral inflammatory infiltrate, alongside MMR status determined through MSH6 and PMS2 markers. PD-L1 positivity (evaluated using Rabbit Anti-Human Monoclonal Antibody (clone SBC-992, Sina Biotech and defined as expression above 1% in tumor cells and over 5% in inflammatory cells)) was detected in 57.15% and 39.28% of cases, respectively. Of the four OCCC samples with MMR deficiency, three also expressed PD-L1, suggesting a possible link between defective DNA repair and a more immunogenic tumor profile. One case showed strong PD-L1 positivity (>50%) in tumor cells, along with complete loss of MSH6 and MSH2 expression [22].

**Figure 1:**
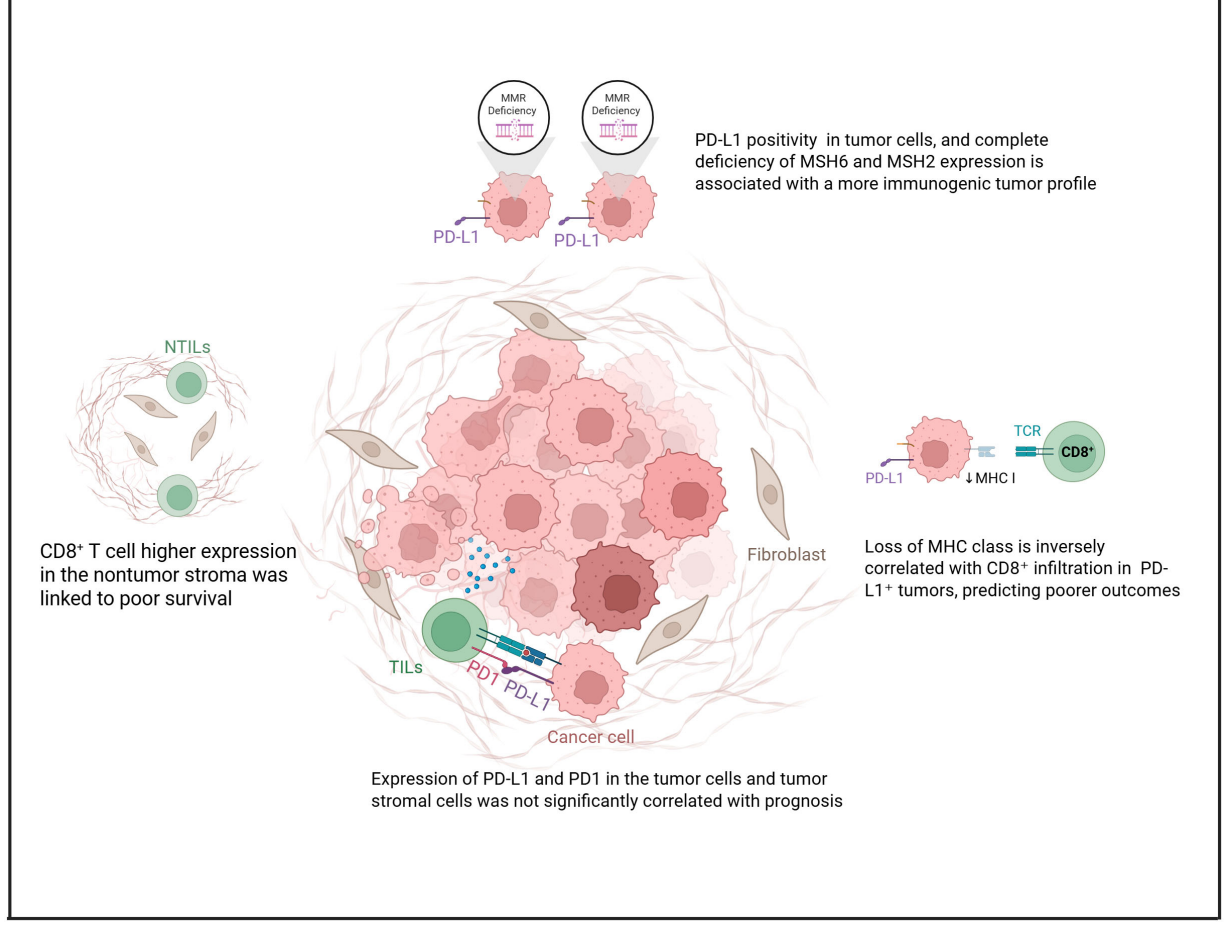
Role of PD-L1 in the tumor microenvironment of OCCC concerning MHC-I loss, TILs, and MMR status. Created in https://www.biorender.com/ (Capella, G. (2025)).

Emerging evidence points toward the existence of a biologically distinct subset of OCCCs that exhibit microsatellite instability (MSI). Unlike the broader OCCC population—typically resistant to conventional chemotherapy—MSI-positive tumors appear markedly more immunogenic. A study performed in the USA on 30 OCCC has shown these tumors to be densely infiltrated by CD8 and PD-1 positive T cells, alongside consistent PD-L1 expression (evaluated with E1L3N clone, the positivity was defined as greater than or equal to 5% of tumor cells with PD-L1 positivity) across both neoplastic and immune compartments. This sets them apart not only from microsatellite-stable OCCC but also from HGSOCs, which generally display a less-activated immune profile [23]. Interestingly, the immune features of MSI-OCCCs resemble those of MSI-positive colorectal and endometrial cancers, tumor types that have demonstrated robust responses to ICI [23]. Figure 1 summarizes the role of PD-L1 within the TIME of OCCC. These divergent prognostic associations—where some immune checkpoints are linked to both favorable and unfavorable outcomes—reflect the heterogeneity of study designs, cohort characteristics, antibody clones, scoring systems (e.g., TPS vs. CPS), and cut-off thresholds. Explicitly acknowledging these methodological differences helps reconcile the apparent contradictions and underscores the need for standardized approaches in future investigations.

## 3. Genes and Pathways Related to Tumor Immune Microenvironment

Activation of oncogenes, such as MYC, RAS, and phosphatidylinositol 4,5-bisphosphate 3-kinase catalytic subunit alpha (PIK3CA), or inactivation of tumor suppressor genes, including TP53, phosphatase and tensin homolog (PTEN), and ARID1A, contributes to TIME. These genetic alterations modulate immune cell infiltration and function by affecting cytokine signaling, antigen presentation, and interferon (IFN) pathways [24].

### 3.1. KRAS and MYC

**Figure 2:**
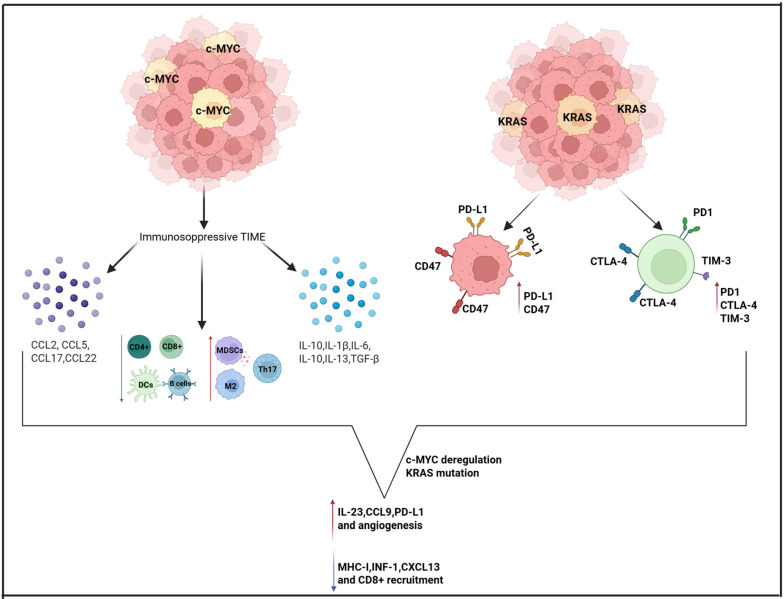
Interplay between MYC, KRAS, and immunosuppression in OCCC. Created in https://www.biorender.com/ (Capella, G. (2025)).

Oncogenic KRAS plays a pivotal role in immune evasion by driving the upregulation of key immune checkpoint ligands, notably PD-L1 and CD47, on the surface of tumor cells. This molecular profile is associated with the upregulation of exhaustion markers—PD-1, Cytotoxic T-Lymphocyte-Associated Protein 4 (CTLA-4), and TIM-3—on T cells, ultimately impairing their cytotoxic function and promoting immune evasion by the tumor [25]. Concurrently, deregulated MYC orchestrates a profoundly immunosuppressive TIME. It stimulates the production of a variety of cytokines (such as transforming growth factor beta (TGF-β), IL-1β, IL-6, IL-10, and IL-13), chemokines (including CCL2, CCL5, CCL17, and CCL22), metabolic byproducts like lactic acid, and other regulatory proteins such as TWIST1 and aurora kinase (AURKA). These factors downregulate antigen presentation (via MHC-I inhibition), dampen IFN signaling pathways (e.g., IFN-I, CXCL13, STAT1/2), and ultimately impair the activity and recruitment of CD8^+^ and CD4^+^ T cells, natural killer (NK) cells, dendritic cells, and B cells. At the same time, they promote the infiltration of immunosuppressive cellular populations, including M2 macrophages, MDSCs, regulatory T cells (Tregs), Th17 cells, mast cells, and endothelial cells [25]. Notably, PD-L1, CD47, and CD276 are also frequently overexpressed in these MYC-driven tumors, reinforcing their immune-resistant phenotype. When KRAS mutations co-occur with MYC deregulation, these tumors exhibit an even more aggressive immunoevasive profile. They upregulate IL-23, CCL9, PD-L1, and CD47 while concurrently suppressing MHC-I, IFN-I, and CXCL13 expression [25]. This dual-axis alteration reduces CD8^+^ T cell cytotoxicity, limits effector cell recruitment, and promotes angiogenesis via vascular endothelial growth factor VEGF-expressing M2 macrophages, driving tumor progression and resistance to immune clearance [25]. K-RAS is a marker of poor prognosis and chemoresistance in OC and can be expressed in up to 25.9% of OCCC [26]. Interestingly, MYC has also been observed to be overexpressed in OCCC and is associated with a poorer prognosis [27,28,29]. Using the ID8 murine OC cell line transduced with c-MYC or KRAS, researchers found that both oncogenes markedly accelerated tumor progression in vivo, despite no increased proliferation in vitro. KRAS expression, in particular, caused rapid ascitic fluid buildup within 2–3 weeks post-injection—much earlier than in control or c-MYC cases. While ascitic VEGF levels were elevated in the c-MYC-driven tumors, KRAS-driven disease was associated with higher concentrations of inflammatory cytokines and an increased presence of neutrophils. Furthermore, a cytokine profiling array identified a marked upregulation of granulocyte–macrophage colony-stimulating factor (GM-CSF) in KRAS-transduced cells. These findings support the notion that, beyond their intrinsic proliferative roles, oncogenes such as MYC and KRAS critically shape the TME, ultimately facilitating tumor progression through distinct immunomodulatory pathways [30]. These findings suggest that, even in OCCC, an interplay between MYC and KRAS may influence the immune response, similar to that observed in other malignancies. Figure 2 summarizes the role of MYC and KRAS within the TIME of OCCC.

### 3.2. PI3K

Phosphoinositide 3-kinase (PI3K) activation orchestrates key intracellular signaling events that regulate immune cell survival, metabolism, and differentiation. In immune contexts, PI3K signaling—particularly via the Akt/mTOR axis—enhances the stability and suppressive function of Tregs by promoting Foxp3 expression and metabolic regulations [31,32,33]. Concurrently, PI3K activity fosters the expansion and immunosuppressive phenotype of myeloid-derived suppressor cells (MDSCs), facilitating the secretion of inhibitory mediators such as IL-10, TGF-β, and reactive oxygen species [31,33]. These effects collectively contribute to the establishment of an immunosuppressive TIME, impairing antigen presentation and effector T cell infiltration [32]. Isoform-specific roles, such as PI3Kγ in myeloid cells and PI3Kδ in lymphocytes, further refine this immunomodulation, with PI3Kγ inhibition shown to reprogram suppressive macrophages toward a pro-inflammatory state [33]. Targeting the PI3K signaling pathway has increasingly gained attention as a compelling approach to overcome tumor-mediated immune escape and boost the effectiveness of immunotherapy [34,35].

**Figure 3:**
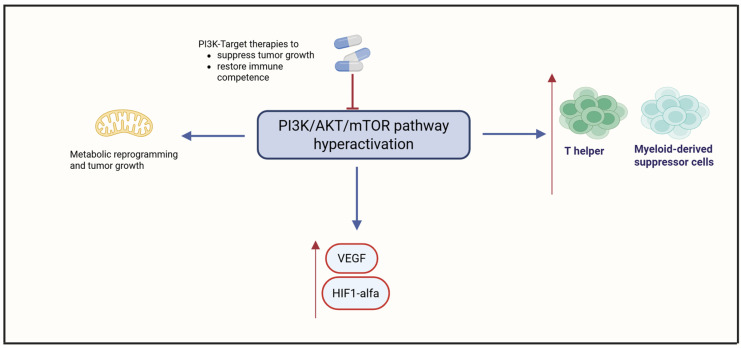
Hyperactivation of the PI3K/AKT/mTOR pathway in OCCC. Created in https://www.biorender.com/ (Capella, G. (2025)).

The PI3K/AKT/mTOR signaling cascade is frequently hyperactivated in OCCC, driving tumor cell survival, unchecked proliferation, and extensive metabolic reprogramming by regulating lipogenesis and glucose metabolism [36,37,38]. This signaling cascade also promotes angiogenesis via upregulation of VEGF and Hypoxia-Inducible Factor 1-alpha (HIF-1α), fostering a hypoxic and immunosuppressive TIME [39]. PI3K activation has been shown to impair dendritic cell maturation and enhance recruitment of Treg cells and MDSCs, thereby dampening antitumor immunity [40]. Dual inhibition of PI3K and mTOR using agents like WX390 has demonstrated synergistic effects with cisplatin in preclinical models, enhancing apoptosis and reversing chemoresistance [40,41]. These findings underscore the potential of PI3K-targeted therapies not only to suppress tumor growth but also to restore immune competence in OCCC, positioning PI3K as a promising therapeutic axis in this challenging malignancy. Figure 3 summarizes the role of PI3K.

### 3.3. P53

The tumor suppressor gene TP53, commonly referred to as p53, plays a pivotal role in safeguarding genome integrity by regulating DNA replication stress and coordinating DNA repair mechanisms [42,43,44]. p53 is activated in response to various cellular stressors, including DNA damage, oxidative stress, and oncogenic signals [45,46]. Upon activation, p53 induces cell cycle arrest and apoptosis to eliminate cells harboring irreparable damage. Notably, p53 also modulates cellular metabolism, establishing a complex interplay that influences the survival or death of tumor cells [47,48]. The tumor suppressor p53 also plays a pivotal role in shaping the immune landscape within tumors [49]. Its mutation or deletion fosters an immunosuppressive TIME through various mechanisms. Loss or mutation of p53 impairs type I IFN production, leading to reduced infiltration of CD4^+^ T cells, CD8^+^ T cells, and NK cells. Moreover, mutant p53 promotes the exhaustion of CD8^+^ T cells via IL-17 signaling and synergizes with TGF-β to drive epithelial–mesenchymal transition (EMT) in colorectal cancer, thereby enhancing metastatic potential [50]. These changes are further reinforced by increased IFNγ-mediated expression of immune checkpoint ligands PD-L1 and PD-L2, a phenomenon that has also been documented in melanoma [50]. In the absence of functional p53, tumor cells exhibit upregulated secretion of WNT ligands, CXCL1, CCL3, CCL21, and miR-149–3p, promoting the differentiation of immunosuppressive MDSCs types such as tumor-associated macrophages and Tregs [50]. In pre-malignant epithelial contexts, p53 deficiency stimulates release of IL-6 and IL-8, facilitating EMT and tumor initiation [50]. Conversely, wild-type p53 acts as an immune enhancer. In various cancer types, it promotes MHC-I expression, antigen presentation, and the transcription of immunostimulatory genes, including TRAIL, DR5, TLRs, PKR, and ULBP1/2 [51]. Through STING activation, p53 supports the cGAS–STING pathway, inducing growth arrest and apoptosis. Interestingly, its regulation of PD-L1 expression appears context-dependent, varying by cancer type [51]. Mutant or deleted p53 disrupts these pro-immunogenic pathways. Such alterations suppress MHC Class I and II expression, impair IRF3 activity, and elevate NF-κB signaling, including IL-6-driven STAT3 phosphorylation [51]. Additionally, p53-deficient tumors escape immune surveillance by downregulating TRAIL receptors and upregulating PD-L1 [51]. In preclinical models, p53 mutations also reduce NK cell ligands—like NKG2D ligands, PVR, and Nectin-2—and impair the cGAS-STING pathway [51]. Mutant p53 also downregulates key apoptotic mediators (e.g., NOXA, BAX, and PUMA), rendering tumor cells resistant to NK-mediated killing [52]. Moreover, p53-mutant cells secrete PD-L1-bearing extracellular vesicles that inhibit macrophage phagocytosis, while alterations in their secretome (including reduced IL-15 and elevated TGF-β) further suppress the antitumor immune response [52].

HGSOC is characterized by an exceptionally high frequency of TP53 mutations, present in over 95% of cases [53]. These mutations—primarily missense or nonsense—lead to either aberrant accumulation or complete loss of p53 protein, disrupting its role in cell cycle regulation, DNA repair, and apoptosis [53]. p53 dysfunction promotes genomic instability—a hallmark of HGSOC—and drives tumor progression and chemotherapy resistance. Immunohistochemical analysis reliably reflects mutation status; diffuse nuclear overexpression usually indicates missense mutations, while complete absence suggests nonsense or frameshift variants [54]. Given TP53’s immunomodulatory role and its high mutation rate in HGSOC, TP53 status likely shapes the tumor’s immune landscape in this cancer type.

**Figure 4:**
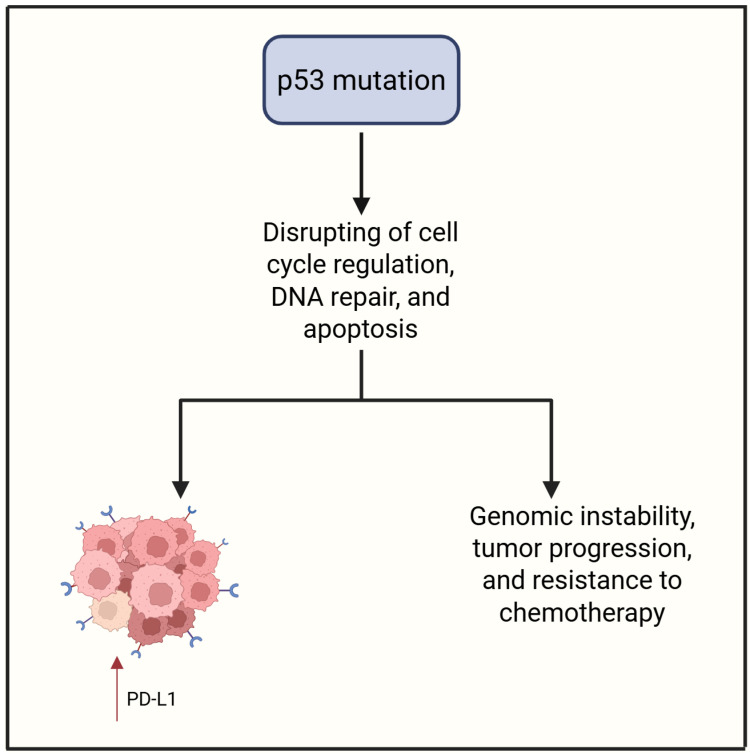
p53 mutation contributes to PD-L1 expression and aggressive tumor features. Created in https://www.biorender.com/ (Capella, G. (2025)).

Regarding OCCC, *TP53* mutations appear to be significantly less frequent compared to HGSOC [55,56,57,58]. For instance, in a cohort of 38 OCCC cases, aberrant p53 expression was observed in only a small subset, with confirmed mutations—including missense variants and small deletions—identified in just four tumors. These findings suggest that p53 alterations may not play a central role in the pathogenesis of OCCC [57,59,60]. Aberrant p53 expression has been linked to poor clinical outcomes, including reduced OS and higher recurrence risk [61]. Recent studies also associate p53 abnormalities in OCCC with features like tumor budding and PD-L1 expression, suggesting a role in shaping the TIME [61]. Given p53’s immunomodulatory functions and prognostic value in HGSOC, TP53 status may also affect immune dynamics in OCCC, though likely in a more limited or context-specific way. These insights suggest that TP53 status may serve not only as a prognostic marker but also as a potential determinant of immunotherapy responsiveness, warranting further investigation into p53-guided therapeutic strategies in ovarian cancer. Figure 4 summarizes the action of the p53 mutation.

### 3.4. PTEN

Somatic loss or inactivation of PTEN leads to aberrant activation of the PI3K/AKT/mTOR signaling pathway, which governs cellular proliferation, survival, and metabolic processes [24,62,63,64]. In preclinical research, PTEN loss is strongly associated with aggressive tumor phenotypes [65] and also contributes to the establishment of an immunosuppressive TIME [66,67,68]. In mouse models, this immune landscape is characterized by impaired T cell infiltration and reduced antitumor immune responses, undermining the efficacy of immunotherapies and promoting tumor immune evasion [69].

In preclinical models of various cancer types, loss of PTEN leads to the accumulation of immunoregulatory cells—including MDSCs, Tregs, and M2-polarized macrophages—which collectively inhibit cytotoxic immune activity [70]. Tumors with PTEN loss also show upregulation of immune checkpoint molecules such as PD-L1 and the immunosuppressive enzyme IDO1 [70]. Paradoxically, in pan-cancer analysis, nuclear PTEN loss has been associated with increased genomic instability and neoantigen generation, potentially enhancing tumor immunogenicity [70]. Even in this context, the immunosuppressive environment seems to dominate, allowing tumor cells to escape immune detection despite a potentially more antigenic landscape [70]. PTEN mRNA expression (evaluated with xCell, a web-based algorithm that estimates the relative abundance of immune and stromal cell types using bulk-tissue RNA-seq profiles) shows a positive correlation with CD4/CD8A gene expression and effector T cell infiltration—particularly helper, central memory, and effector memory subsets—across multiple tumor types [71]. Conversely, genomic deletion of PTEN correlates with reduced CD8^+^ and Th1 cells, increased Th2 polarization, and elevated expression of immunosuppressive genes such as *VEGFA*. Tumors harboring PI3K pathway activation, including *PIK3CA* or *PIK3CB* amplification, exhibit a T cell-excluded phenotype similar to that seen in cancers with PTEN loss [71]. Overall, PTEN loss and downstream PI3K signaling are linked to reduced responsiveness to immunotherapy and poorer clinical outcomes [71].

**Figure 5:**
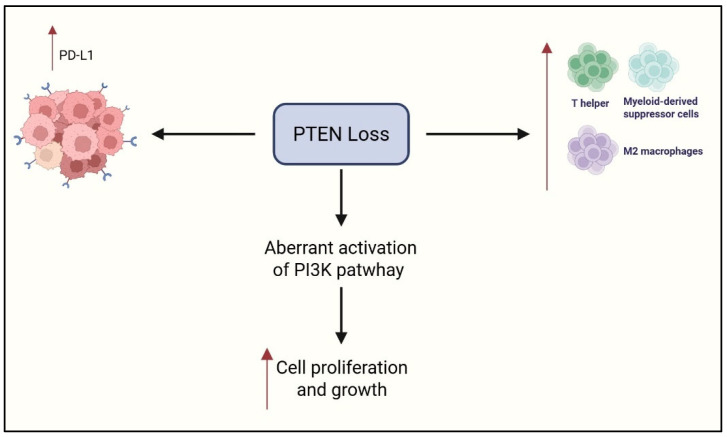
Role of PTEN loss in immune response and cancer growth. Created in https://www.biorender.com/ (Capella, G. (2025)).

Regarding OCCC, approximately 40% of these tumors harbor mutations or deletions affecting the *PTEN* gene [72,73,74]. PTEN loss demonstrates divergent associations with CD8^+^ TIL density in different OC subtypes [75]. In HGSOC, reduced PTEN expression correlates with lower CD8^+^ T cell counts and is notably linked to longer OS (HR = 0.78; 95% CI: 0.65–0.94; *p* = 0.022), suggesting a nuanced role in modulating immune surveillance. This counterintuitive association may reflect the genomic instability of HGSOC, where PTEN loss often co-occurs with chromosomal copy number alterations and homologous recombination deficiency, leading to activation of DNA damage response pathways (e.g., ATM) and cytokine-mediated recruitment of cytotoxic T cells—ultimately contributing to improved survival outcomes despite lower PTEN expression. Conversely, OCCC PTEN loss is associated with higher CD8^+^ infiltration (*p* < 0.0001), supporting the hypothesis that PI3K pathway dysregulation may influence cytotoxic T cell recruitment differently across histological subtypes. Cytoplasmic PTEN downregulation is most frequently observed in endometrioid and OCCC—particularly in younger endometrioid OC patients (*p* = 0.0001). PTEN expression also shows significant associations with hormone receptor status (ER: *p* = 0.0008; PR: *p* = 0.062; AR: *p* = 0.0002) in HGSOC and with CD8^+^ density patterns in advanced-stage disease, where heterogeneous PTEN expression corresponds to increased CD8^+^ TIL counts (*p* = 0.0016) [75]. These findings highlight PTEN’s immunomodulatory role and its context-dependent link to the CD8^+^ compartment in OC. In OCCC, PTEN loss is strongly associated with PD-L1 expression (*p* = 0.007) [76]. Immunohistochemistry showed that all PD-L1^+^ tumors had concurrent PTEN loss, suggesting that PTEN disruption may drive immune checkpoint upregulation. PTEN influences tumor immunogenicity by regulating transcriptional programs affecting PD-L1 expression [75]. This mechanism suggests that PTEN inactivation promotes immune suppression via PD-L1 induction, revealing immunological vulnerabilities in OCCC and guiding targeted immunotherapy strategies [76]. Furthermore, PTEN loss was associated with worse PFS in early-stage disease (*p* = 0.039), underscoring its prognostic relevance and therapeutic potential in the context of ICI [76]. Figure 5 summarizes the role of PTEN.

### 3.5. ARID1A

ARID1A, encoded by the *ARID1A* gene, is an integral component of the canonical BAF (BRG1-associated factors) complex, one of the three distinct mammalian SWI/SNF (Switch/Sucrose Non-Fermentable) chromatin remodeling assemblies, alongside PBAF (polybromo-associated BAF) and ncBAF (non-canonical BAF). These SWI/SNF complexes are pivotal in regulating key cellular processes, including differentiation, proliferation, and DNA repair [77].

Within the cBAF complex, DNA binding is mediated through either ARID1A or its mutually exclusive paralog ARID1B [78]. Dysregulation of mSWI/SNF complexes has been recurrently implicated in tumorigenesis and various neurodevelopmental disorders [79]. Among the subunits, ARID1A represents the most frequently mutated gene across diverse cancer types [79].

ARID1A mutations are present in approximately 30–57% of OCCC, typically manifesting as nonsense or frameshift deletions that produce truncated, non-functional protein products [80,81]. Interestingly, even monoallelic inactivation can result in complete loss of ARID1A expression [82], underscoring its vulnerability as a tumor suppressor gene. In one cohort, ARID1A mutations were detected in 41.5% of OCCC cases, while ARID1A protein loss reached 75.6% [83]. Notably, ARID1A mutations in OCCC were tightly linked to immunogenic features, including elevated PD-L1 and CD8^+^ T cell expression (*p* < 0.001) and high tumor mutational burden (TMB; *p* = 0.006). Loss of ARID1A expression similarly correlated with PD-L1 upregulation and CD8 infiltration in OCCC and HGSOC, but not in endometrioid or mucinous tumors, suggesting subtype-specific immune modulation [83]. These associations identify ARID1A as both a molecular hallmark of CCC and a potential biomarker for immune checkpoint blockade response. Double-mutant ARID1A CCC tumors consistently showed PD-L1 positivity (≥1%) and stromal CD8^+^ infiltration in about 77% of cases—features of a ‘hot’ immune-inflamed microenvironment.

Beyond surface markers, ARID1A-mutated tumors exhibit baseline immune activation, characterized by enrichment of CXCL13^+^ CTLA4^+^ neoantigen-reactive CD8^+^ T cells and heightened FASLG–FAS signaling [84]. However, recurrent OCCC adopts a fibrotic and angiogenic phenotype, with metabolic reprogramming toward fatty acid utilization and immunosuppressive features, including upregulation of CD36 and CD47—both associated with worse clinical outcomes. Targeting VEGF via bevacizumab enhances intratumoral T cell infiltration and IFN-γ signaling, and combined blockade of VEGF and PD-1 pathways has shown therapeutic promise in persistent and metastatic OCCC [84]. Moreover, tumor–stroma interactions involving POSTN and metabolic mediators reinforce the role of the microenvironment in therapy resistance and disease progression [84].

**Figure 6:**
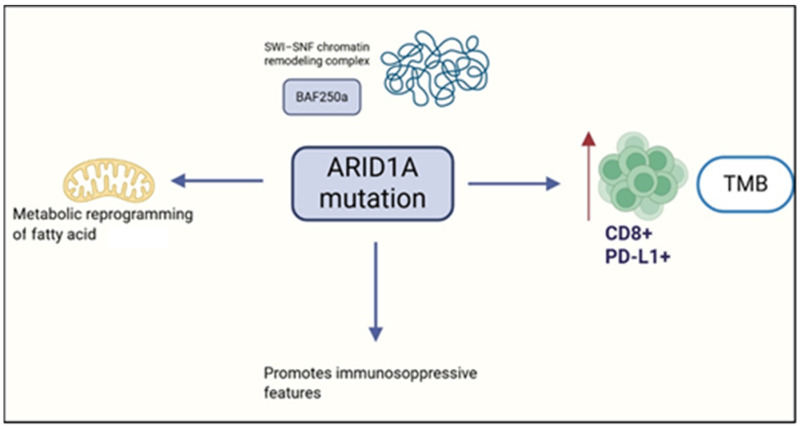
Role of ARID1A on the tumor immune environment. Created in https://www.biorender.com/ (Capella, G. (2025)).

Spatial and immunophenotypic characterization of OCCC biopsies from 45 patients (early vs. advanced FIGO stages) further underscores the complexity of the TIME [85]. Using 14 immune markers, PD-1 ligands, collagen profiling, and transcriptomic analysis, the study revealed distinct immune landscapes between ARID1A-wild-type and mutant tumors. CD138^+^ plasma cells were enriched in the tumor core, CD20^+^ B cells and T cells localized at the invasive front, and mast cells dominated the stroma. PD-L2 was the main PD-1 ligand, concentrated on malignant cells. Advanced-stage OCCCs showed dense fibroblast networks and complex collagen structures, with TGFβ-driven remodeling as a key pathway. Notably, CD8^+^ infiltration in tumor cores and CD4^+^ presence in peripheral and stromal zones were independently linked to reduced OS [85]. These findings suggest that the progressive remodeling of the collagen matrix—from the malignant cell area through the leading edge into the stroma—creates a spatially complex and heterogeneous extracellular matrix that may hinder immune cell infiltration and facilitate immune evasion. The increased fiber density, branching, and texture complexity in the stroma could act as physical and biochemical barriers, contributing to resistance against immune-mediated clearance. Figure 6 summarizes the role of ARID1A.

## 4. Natural Killer Cell Impairment

NK cell dysfunction was characterized in patients with chemoresistant OCCC. Analysis of NK cells from peripheral blood (PB) and peritoneal fluid (PF) showed that PF-derived NK cells had significantly reduced degranulation when co-cultured with autologous tumor cells (ATCs) [86]. Tumor ligand analysis showed that ATCs maintained a consistent HLA class I^+^ phenotype across patients, albeit with variable expression intensity, and displayed heterogeneous levels of ligands for NK-activating receptors. Crucially, tumor-infiltrating NK cells exhibited downregulation of key activating receptors, predominantly DNAX Accessory Molecule-1 (DNAM-1), suggesting a tumor-induced suppressive TIME. These data support a dual mechanism underlying the defective cytotoxic activity: (i) enhanced inhibitory signaling via HLA-I–specific NK receptors, and (ii) insufficient engagement of activating receptors due to ligand loss or alteration. This immunoevasive profile may underlie OCCC’s resistance to conventional therapies and inform the development of NK cell-based immunotherapy strategies [86].

## 5. Immune Checkpoint Inhibitors in Ovarian Clear Cell Carcinoma

ICIs have revolutionized the treatment landscape for multiple solid tumors, including non-small cell lung cancer, melanoma, bladder cancer, endometrial and cervical cancers, and renal cell carcinoma. Despite compelling preclinical data advocating their use in EOC, early-phase trials have largely underperformed [11]. Nevertheless, a subset of patients with recurrent OCCC appears to derive notable benefit from ICIs, as suggested by emerging clinical data [87,88].

Among the pivotal investigations, the JAVELIN Ovarian 200 trial—a randomized, three-arm phase III study—evaluated avelumab as monotherapy or in combination with pegylated liposomal doxorubicin (PLD) versus PLD alone in platinum-resistant or platinum-refractory EOC. Seventy-three patients with OCCC were included [89]. In this subgroup, treatment with avelumab plus PLD resulted in a median PFS of 2.8 months (Hazard Ratio—HR: 0.79; 95% confidence interval—CI: 0.41–1.54), and a median OS of 17.7 months (HR: 0.89; 95% CI: 0.38–2.06).

Complementing these findings, the PEACOCC trial—a multicenter, single-arm phase II study—assessed pembrolizumab monotherapy in advanced clear cell gynecological cancers (CCGCs), predominantly of ovarian origin (85%) [90]. Despite a high rate of mismatch repair proficiency (98%), the 12-week PFS was 42%, and the objective response rate (ORR) reached 25%, albeit exclusively partial responses. Though the median PFS was modest (2.7 months), an OS of 14.8 months highlighted durable responses in select individuals.

In contrast, the MOCCA phase II trial compared durvalumab to the physician’s choice of chemotherapy in recurrent OCCC [91]. While durvalumab did not demonstrate statistically significant advantages—median PFS of 7.6 weeks vs. 14.0 weeks (HR = 1.6; *p* = 0.92) and median OS of 37.9 vs. 40.6 weeks (HR = 1.5; *p* = 0.85)—exploratory analysis hinted at biomarker-driven response patterns. PD-L1 positivity (CPS ≥ 1%) was seen in 28.9% of cases, and PIK3CA mutations were associated with improved disease control (Relative Risk—RR = 2.83; 95% CI: 1.16–14.17).

Dual checkpoint blockade strategies are increasingly explored. A randomized phase II study evaluated nivolumab (anti–PD–1) alone versus its combination with ipilimumab (anti-CTLA–4) in relapsed CCGCs [92]. Among 44 treated patients (82% ovarian), the combination arm achieved an ORR of 33% (including four complete responses), outperforming nivolumab alone (ORR 14.3%). Median PFS was 5.6 vs. 2.2 months, and OS was 24.6 vs. 17 months, respectively.

Further evidence of durable benefit emerged from the SWOG S1609 DART trial evaluating nivolumab and ipilimumab in rare CCGCs [93]. Among 19 patients with OCCC, the ORR was 21.1% with three complete responses—two ongoing beyond three years—and a clinical benefit rate (CBR) of 31.6%. Median PFS and OS were 3.7 and 21.7 months, respectively.

The MoST-CIRCUIT trial [94] similarly explored dual ICI (nivolumab plus ipilimumab) in advanced OCCC (N = 24) and uterine carcinomas (N = 4). ORR reached 50% across both subgroups, including 13% complete and 37% partial responses. Six-month PFS was 52%, and median OS was 9.9 months. Interestingly, the response correlated with higher TMB, and ARID1A wild-type status appeared predictive.

### Combination Between ICI and Other Target Therapies

Given the limited monotherapy efficacy of ICIs in OCCC, multiple trials have pivoted toward combination strategies. The INOVA trial assessed sintilimab (PD-1 inhibitor) combined with bevacizumab (anti-VEGF) in patients with relapsed or persistent OCCC. Among 37 evaluable patients, the ORR was 40.5%, with five complete responses. Median PFS and OS were 6.9 months (95% CI: 5.3–8.1) and 28.2 months (95% CI: 23.8–NR), respectively [95].

Building on immune–metabolic synergy, NRG-GY016 investigated pembrolizumab with epacadostat (IDO1 inhibitor) in 14 patients with OCCC [96]. Though preliminary, results indicated a median PFS of 4.8 months (95% CI: 1.9–9.6) and an ORR of 21% [96].

The LARA study offered compelling evidence for the combination of pembrolizumab and lenvatinib (a multi-targeted tyrosine kinase inhibitor) in recurrent CCGC. Of 25 evaluable patients, 44.0% achieved confirmed responses within 24 weeks (95% CI: 24.4–65.1), and median PFS reached 23.4 weeks (95% CI: 4.4–42.4) [97].

Ongoing trials continue to refine these strategies. The DOVE/APGOT-OV7/ENGOT-ov80 study is a randomized phase II trial comparing dostarlimab ± bevacizumab with single-agent chemotherapy [98]. Additionally, the camrelizumab–anlotinib combination (NCT05600998) represents another active investigation into dual immunotherapy-targeted agent platforms, reinforcing the expanding paradigm of combination-based immunotherapy in OCCC [9]. An overview of the outcomes from trials evaluating ICIs for the treatment of OCCC is provided in Table 1.

## 6. Discussion

The preclinical evidence delineates a multifaceted and immunologically paradoxical TIME in OCCC, where elevated infiltration by cytotoxic and Th cells paradoxically correlates with poorer prognosis, likely due to concurrent mechanisms of immune evasion such as MHC-I downregulation and regulatory cell accumulation [16,20]. PD-L1 expression, while conventionally associated with immune suppression, occasionally demonstrates favorable prognostic value, underscoring a nuanced immunobiological interplay influenced by tumor context and checkpoint saturation [16,17,18]. Genomic alterations—particularly in KRAS, MYC, PI3KCA, TP53, PTEN, and ARID1A—act as central modulators of TIME, driving checkpoint ligand overexpression (e.g., PD-L1, CD47), suppression of antigen presentation, expansion of immunosuppressive subsets (Tregs, MDSCs, M2 macrophages), and disruption of IFN signaling [24,25,26,27,28,29,30,50,51,52,70,83]. KRAS/MYC co-alteration amplifies immunoevasive phenotypes through synergistic cytokine and metabolic dysregulation [25,26,27,28,29,30], while PI3K pathway hyperactivation facilitates angiogenesis and immune suppression via VEGF/HIF-1α upregulation [36,37,38,39,40]. PTEN loss serves as both a driver of immunosuppressive remodeling and a facilitator of PD-L1 induction [70,76], further impairing cytotoxic lymphocyte infiltration. ARID1A deficiency defines a potentially immunogenic molecular subset of OCCC, characterized by high TMB, stromal CD8^+^ infiltration, and PD-L1 positivity—suggesting ARID1A as a candidate biomarker for ICI responsiveness, although further clinical validation is needed [83,84,85]. Conversely, TP53 mutations—though less prevalent in OCCC than in HGSOC—when present, contribute to TIME dysregulation through IFN-I suppression and the upregulation of immune checkpoint ligands [50,51,52,61]. NK cell dysfunction, marked by diminished activating receptor signaling and sustained HLA-I expression on tumor cells, further reinforces immune escape mechanisms—likely via engagement of HLA-I-specific inhibitory receptors such as KIRs and NKG2A [86]. Clinically, ICIs as monotherapy have demonstrated modest efficacy in OCCC, with limited PFS and overall response rates [89,90,91]. However, dual checkpoint inhibition—particularly anti-PD-1 plus anti-CTLA-4—achieves superior outcomes, with higher ORR and prolonged survival, even complete responses in some patients [92,93,94]. The integration of ICIs with targeted agents (e.g., anti-VEGF, IDO1 inhibitors, multi-TKI) significantly enhances therapeutic efficacy, with ORRs up to 50% and sustained disease control in biomarker-selected populations [95,96,97]. Biomarkers, including PD-L1 CPS ≥ 1%, ARID1A mutations, PIK3CA mutations, and elevated TMB, are under investigation as potential predictors of ICI responsiveness. However, their predictive value remains to be confirmed in prospective clinical trials. In contrast, PTEN loss and MMR deficiency may modulate immunogenicity in subtype-specific manners [76,83,84,85,91,92,93,94]. These findings support a biomarker-driven approach to immunotherapy in OCCC, emphasizing the need for patient selection based on emerging molecular features (e.g., ARID1A mutations, PD-L1 CPS ≥ 1%, elevated TMB) and rational combination regimens (e.g., dual checkpoint blockade, ICIs plus anti-VEGF or multi-TKIs), while acknowledging the preliminary nature of current evidence and the necessity for further clinical validation. The variable clinical responsiveness of OCCC to ICIs may stem from its uniquely immunosuppressive tumor microenvironment, where paradoxical immune cell infiltration coexists with profound evasion mechanisms. Translational challenges include accurately modeling this complex TIME in preclinical systems and identifying reliable biomarkers that capture dynamic immunogenic shifts, which remain critical for optimizing immunotherapy strategies

## 7. Conclusions

In summary, OCCC presents a biologically distinct and therapeutically challenging landscape—characterized by an immunologically active yet functionally suppressed TIME. While conventional checkpoint inhibition shows limited benefit, emerging data highlight the value of combinatorial strategies and biomarker-driven approaches in unlocking durable immune responses. Harnessing genomic insights and refining patient selection will be key to transforming immunotherapy from an exploratory option into a validated standard within this rare and aggressive subtype.

## Figures and Tables

**Table 1:** Published results of trials evaluating ICC in the treatment of OCCC.

Reference	Drug	Study Design	Clinical Setting	Number of Patients	Biomarkers	Main Results
Hamanishi et al. [86]	Nivolumab	Phase II	Platinum-resistant EOC	26 EOC (2 OCCC)	/	1 CR out of 2 cases with OCCC
KEYNOTE-100 [88]	Pemrbolizumab	Phase II	Recurrent, heavily pretreated EOC	285 EOC (2 OCCC)	/	1 CR out of 2 cases with OCCC
JAVELIN Ovarian 200 [89]	Avelumab or avelumab + PLD or PLD	Phase III	Platinum-resistant or refractory EOC	566 EOC (73 OCCC)	/	PFS: 2.8 months (HR: 79; 95% CI 0.41–1.54), OS: 17.7 months (HR: 0.89; CI: 0.38–2.06)
PEACCOC trial [90]	Pembrolizumab	Phase II	Advanced CCGC	48 CCGC (41 OCCC)	MMR, ARID1A, p53, PD-1, PD-L1 (none predictive of treatment response)	ORR: 25%. PFS 2.7 months, OS: 14.8 months
MOCCA trial [91]	Durvalumab vs. CHT	Phase II	Recurrent OCCC	48 OCCC	ARID1A, PIK3CA, KRAS, TERT, PD-L1, PI3KCA (associated with long time to progression), ERBB2 (associated with worse survival)	PFS: 7.6 weeks (95% CI 7.0–16.0), OS: 37.9 weeks (95% CI 21.7–143.0)
BrUOG 354 [92]	Nivolumab or nivolumab + ipilimumab	Phase II	Recurrent extra-renal CCC	44 CCC (36 OCCC)	/	ORR of 33% for nivolumab/ipilimumab versus 14.3% with nivolumab alone. PFS: 5.6 months for nivolumab/ipilimumab and 2.2 months with N; OS: 24.6 months for nivolumab/ipilimumab and 17 months for nivolumab
SWOG S1609 trial [93]	Nivolumab + imiplimab	Phase II	CCGC	19 OCCC	/	ORR: 21.1%. Median PFS: 3.7 months. Median OS: 21.7 months
MoST-CIRCUIT trial [94]	Nivolumab or nivolumab + ipilimumab	Phase II	Advanced OCCC or UCCC	24 OCCC; 4 UCCC	PIK3A, TMB (predictive of response), ARID1A (wild-type predictive of response)	ORR: 50%. PFS rate at 6 months: 52%. Median OS: 9.9 months
Inova trial [95]	Sintilimab + bevacizumab	Phase II	Recurrent/persistent OCCC	41 OCCC	TMB, MMR, PD-L1, TILs, ARID1A (none predictive of treatment response)	ORR: 40.5%. Median PFS: 6.9 months (95% CI 5.3–8.1); median OS: 28.2 months (95% CI 23.8–NR)
NRG-GY016 trial [96]	Pembrolizumab + epacadostat	Phase II	Recurrent OCCC	14 OCCC	/	ORR: 21%. PFS: 4.8 months (95% CI: 1.9–9.6)
LARA trial [97]	Pembrolizumab + lenvatinib	Phase II		25 OCCC		ORR: 44.0%. PFS: 23.4 weeks (95% CI: 4.4–42.4)

ARID1A: AT-rich interaction domain 1A; CCGC: clear cell gynecological cancer; CHT: chemotherapy; CI: confidence interval; CR: complete response; ECCC: endometrial clear cell carcinoma; EOC: epithelial ovarian cancer; MMR: mismatch repair; NR: not reached; OCCC: ovarian clear cell carcinoma; ORR: objective response rate; OS: overall survival; PD-L1 programmed death-ligand 1; PFS: progression-free survival, PIK3CA: phosphatidylinositol-4,5-bisphosphate 3-kinase catalytic subunit alpha; PLD: pegylated liposomal doxorubicin; TERT telomerase reverse transcriptase; TILs: tumor-infiltrating lymphocytes; TMB: tumor mutational burden.

## Abbreviations

**ARID1A**—AT-rich interaction domain-containing protein 1A; **ATC**—Autologous Tumor Cells; **AURKA**—Aurora Kinase A; **BAF**—BRG1-associated factors; **CBR**—Clinical Benefit Rate; **CCGCs**—Clear Cell Gynecological; **CI**—Confidence Interval; **CPS**—Combined Positive Score; **CTLA-4**—Cytotoxic T-Lymphocyte-Associated Protein 4; **EMT**—Epithelial–Mesenchymal; **EOC**—Epithelial Ovarian Cancer; **FIGO**—International Federation of Gynecology and Obstetrics; **GM-CSF**—Granulocyte-Macrophage Colony-Stimulating Factor; **HGSOC**—High-Grade Serous Ovarian Carcinoma; **HIF-1α**—Hypoxia-Inducible Factor 1-alpha; **HR**—Hazard Ratio; **ICI**—Immune Checkpoint Inhibitors; **IDO1**—Indoleamine 2,3-dioxygenase 1; **IFN**—Interferon; **MDSCs**—Myeloid-Derived Suppressor Cells; **MHC-I**—Major Histocompatibility Complex Class I; **MMR**—Mismatch Repair; **MSI**—Microsatellite Instability; **ncBAF**—Non-canonical BAF; **NK**—Natural Killer; **OC**—Ovarian Cancer; **OCCC**—Ovarian Clear Cell Carcinoma; **ORR**—Objective Response Rate; **OS**—Overall Survival; **PB**—Peripheral Blood; **PBAF**—Polybromo-associated BAF; **PD-1**—Programmed Death-1; **PD-L1**—Programmed Death-Ligand 1; **PF**—Peritoneal Fluid; **PFS**—Progression-Free Survival; **PI3K**—Phosphoinositide 3-Kinase; **PIK3CA**—Phosphatidylinositol-4,5-bisphosphate 3-kinase catalytic subunit alpha; **PTEN**—Phosphatase and Tensin Homolog; **RR**—Relative Risk; **SWI/SNF**—Switch/Sucrose Non-Fermentable; **TGF-β**—Transforming Growth Factor Beta; **Th cells**—T helper cells; **TIME**—Tumor Immune Microenvironment; **TILs**—Tumor-Infiltrating Lymphocytes; **TPS**—Tumor Proportion Score; **Tregs**—Regulatory T cells; **TMB**—Tumor Mutational Burden; **VEGF**—Vascular Endothelial Growth Factor.
